# Bioactivity of synthetic peptides from Ecuadorian frog skin secretions against *Leishmania mexicana*, *Plasmodium falciparum*, and *Trypanosoma cruzi*

**DOI:** 10.1128/spectrum.03339-23

**Published:** 2024-07-16

**Authors:** Carolina Proaño-Bolaños, Giovanna Morán-Marcillo, Nina Espinosa de los Monteros-Silva, Sebastián Bermúdez-Puga, Mateo A. Salazar, Ailín Blasco-Zúñiga, Sebastián Cuesta, Carolina Molina, Franklin Espinosa, Lorena Meneses, Patricio Rojas-Silva, Sonia Zapata Mena, Fabián E. Sáenz, Miryan Rivera I., Jaime A. Costales

**Affiliations:** 1 Biomolecules Discovery Group, Laboratory of Molecular Biology and Biochemistry, Universidad Regional Amazónica Ikiam, Tena, Ecuador; 2 Department of Biochemical-Pharmaceutical Technology, Faculty of Pharmaceutical Sciences, University of São Paulo, São Paulo, Brazil; 3 Laboratorio de Investigación en Citogenética y Biomoléculas de Anfibios (LICBA), Centro de Investigación para la Salud en América Latina (CISeAL), Pontificia Universidad Católica del Ecuador, Quito, Ecuador; 4 Centro de Investigación para la Salud en América Latina, Pontificia Universidad Católica del Ecuador, Quito, Ecuador; 5 Laboratorio de Química Computacional, Facultad de Ciencias Exactas y Naturales, Pontificia Universidad Católica del Ecuador, Quito, Ecuador; 6 Instituto de Microbiología, Colegio de Ciencias Biológicas y Ambientales, Universidad San Francisco de Quito, Quito, Ecuador; University of São Paulo, São Paulo, Brazil

**Keywords:** anti-microbial peptide, malaria, Chagas, leishmaniasis, anti-parasitic, frog

## Abstract

Chagas disease, leishmaniasis, and malaria are major parasitic diseases disproportionately affecting the underprivileged population in developing nations. Finding new, alternative anti-parasitic compounds to treat these diseases is crucial because of the limited number of options currently available, the side effects they cause, the need for long treatment courses, and the emergence of drug-resistant parasites. Anti-microbial peptides (AMPs) derived from amphibian skin secretions are small bioactive molecules capable of lysing the cell membrane of pathogens while having low toxicity against human cells. Here, we report the anti-parasitic activity of five AMPs derived from skin secretions of three Ecuadorian frogs: cruzioseptin-1, cruzioseptin-4 (CZS-4), and cruzioseptin-16 from *Cruziohyla calcarifer*; dermaseptin-SP2 from *Agalychnis spurrelli*; and pictuseptin-1 from *Boana picturata*. These five AMPs were chemically synthesized. Initially, the hemolytic activity of CZS-4 and its minimal inhibitory concentration against *Escherichia coli*, *Staphylococcus aureus*, and *Candida albicans* were determined. Subsequently, the cytotoxicity of the synthetic AMPs against mammalian cells and their anti-parasitic activity against *Leishmania mexicana* promastigotes, erythrocytic stages of *Plasmodium falciparum* and mammalian stages of *Trypanosoma cruzi* were evaluated *in vitro*. The five AMPs displayed activity against the pathogens studied, with different levels of cytotoxicity against mammalian cells. *In silico* molecular docking analysis suggests this bioactivity may occur via pore formation in the plasma membrane, resulting in microbial lysis. CZS-4 displayed anti-bacterial, anti-fungal, and anti-parasitic activities with low cytotoxicity against mammalian cells. Further studies about this promising AMP are required to gain a better understanding of its activity.

**IMPORTANCE:**

Chagas disease, malaria, and leishmaniasis are major tropical diseases that cause extensive morbidity and mortality, for which available treatment options are unsatisfactory because of limited efficacy and side effects. Frog skin secretions contain molecules with anti-microbial properties known as anti-microbial peptides. We synthesized five peptides derived from the skin secretions of different species of tropical frogs and tested them against cultures of the causative agents of these three diseases, parasites known as *Trypanosoma cruzi*, *Plasmodium falciparum*, and *Leishmania mexicana*. All the different synthetic peptides studied showed activity against one of more of the parasites. Peptide cruzioseptin-4 is of special interest since it displayed intense activity against parasites while being innocuous against cultured mammalian cells, which indicates it does not simply hold general toxic properties; rather, its activity is specific against the parasites.

## INTRODUCTION

Chagas disease, leishmaniasis, and malaria, caused by the protozoan parasites *Trypanosoma cruzi*, *Leishmania* spp., and *Plasmodium* spp., respectively, are among the most important parasitic infections in Latin America and globally (1–3). However, special challenges exist for the development of anti-microbials to treat these tropical infections. In the case of Chagas disease and leishmaniasis, the pharmaceutical industry has traditionally shown little interest (4, 5).

Chagas disease is the most important parasitic disease in Latin America (6), affecting an estimated 7–8 million people (7). In recent years, human migration has caused the disease to extend to non-endemic regions (7, 8). Chronic infection with *T. cruzi* causes irreversible heart or digestive damages, which may lead to disability and even death in ~30% of those infected (9). Only two drugs, benznidazole and nifurtimox, are currently approved for Chagas disease treatment (10), and neither of them is satisfactory because of lack of effectiveness, especially in chronic infections, in addition to their toxicity and side effects (11).

Leishmaniasis is a neglected tropical disease that mainly affects the economically disadvantaged population in different countries in Asia, Africa, the Caribbean, and Latin America (12–15). Current treatment is based on drugs such as miltefosine, amphotericin B, and pentavalent antimonials; the latter two require parenteral administration. These drugs cause significant side effects and must be administered under close medical supervision, which complicates adherence to treatment (12).

Malaria kills more people in the world than any other parasitic disease, and its control and elimination are greatly dependent on effective anti-parasitic drugs (16). Nevertheless, *Plasmodium falciparum*, the most aggressive among the species of the genus capable of infecting humans, has developed resistance to the majority of the anti-malarial drugs in use (16). The current treatment based on artemisinin derivatives is losing efficacy in Southeast Asia, making the search for new anti-malarial drugs urgent.

Amphibian skin secretions have been identified as natural sources of bioactive peptides with anti-viral, anti-bacterial, anti-fungal, anti-parasitic, and anti-proliferative activity (17–21). Due to their activity at low concentration, reduced toxicity, and unique mechanisms of action, anti-microbial peptides (AMPs) have been highlighted as potential candidates for anti-microbial therapy (18). The anti-parasitic activity against *Leishmania* sp., *Plasmodium* sp., and *Trypanosoma* sp. has been evaluated in at least three AMP families known as dermaseptins, phylloseptins, and temporins (18, 20–27).

In this context, testing the activity of novel AMPs against pathogens may yield insights into novel avenues for drug development or reveal intrinsic parasite weaknesses, which could be exploited for therapeutic goals. Cruzioseptins and pictuseptins are recently described peptide families identified in two Ecuadorian amphibian species (*C. calcarifer* and *B. picturata*, respectively) (28, 29). We have previously reported the anti-microbial activity of cruzioseptin-1 (CZS-1) and cruzioseptin-16 (CZS-16) from *Cruziohyla calcarifer* (29, 30) and dermaseptin-SP2 (DRS-SP2) from *Agalychnis spurrelli* (31), and the anti-leishmanial activity of CZS-1 against *Leishmania* (L.) *amazonensis* and *Leishmania* (V.) *braziliensis* (32). Furthermore, we have characterized the anti-bacterial activity of pictuseptin-1 (PTS-1) from *Boana picturata* (28).

Here, we perform an *in silico* structural characterization of five peptides (CZS-1, CZS-4, CZS-16, DRS-SP2, and PTR-1), and we evaluate their activity against protozoan parasites. Additionally, we report the anti-microbial and hemolytic activities of CZS-4 against *Escherichia coli*, *Staphylococcus aureus*, and *Candida albicans*.

## MATERIALS AND METHODS

### Solid-phase peptide synthesis

The following amide peptides, CZS-1: GFLDIVKGVGKVALGAVSKLF-NH_2_, CZS4: GFLDVIKHVGKAALSVVSHLINE-NH_2_, CZS-16: GFLDVLKGVGKAALGAVTHLINQ-NH_2_, DRS-SP2: ASWKVFLKNIGKAAGKAVLNSVTDMVNQ-NH_2_, and PTS-1: GFLDTLKNIGKTVGRIALNVLT-NH_2_, were synthetized by solid-phase strategy (solid-phase peptide synthesis) applying 9-fluorenylmethoxycarbonyl chemistry in a 0.1-mM scale as described previously (28). In short, rink amide 4-methylbenzhydrylamine resin (0.59 meq/g) was employed for C-terminal amidated peptide synthesis using an automatic peptide synthesizer with microwave technology (Liberty Blue, CEM). The molecular mass of synthetic products was confirmed by MALDI TOF MS (Axima Confidence, Shimadzu) in positive reflectron mode using the matrix α-cyano-4-hidroxycinnamic acid (10 mg/mL).

### Synthetic peptide purification

Synthetic peptide purity was determined by reverse-phase high-performance liquid chromatography (RP-HPLC) in Jupiter C_18_ column (5 µm, 300 Å, 250 × 4.6 mm). Fifty µL of peptide (1 mg/200 µL of 99.95% H_2_O/0.05% trifluoroacetic acid [TFA]) was injected in a Waters liquid chromatograph with 2489 detector and 1525 binary HPLC pump. A lineal gradient of 30%–100% solvent B [acetonitrile (ACN)/0.05% TFA] with 1-mL/min flow rate was applied for 65 min. The peak areas and the estimated percentage of purity of each peptide were detected using Empower (v.3) software at 214 and 280 nm.

Peptides were partially purified by Sepacore Flash chromatography system X50 (BUCHI). Peptide aliquots (10 mg/mL) were injected several times using a Reveleris C18 Flash Cartridge (4 g, 12.3 × 6 mm). The elution gradient was 5%–100% solvent B (ACN/0.1% TFA) for 35 min. Detection was set at 214 and 280 nm, and manual collection was performed to obtain 100 mg of each purified peptide, at >95% purity. Due to the difficult separation of some peptides by Flash chromatography, further purification was achieved by RP-HPLC.

### Anti-microbial activity and hemolytic assay of CZS-4

The minimum inhibitory concentration (MIC) of CZS-4 over *Escherichia coli* American Type Culture Collection (ATCC) 25922, *Staphylococcus aureus* ATCC 29213, and *Candida albicans* ATCC 10231 was determined, as previously described (28, 30, 31). In brief, overnight cultures of each microorganism were subcultured in Muller-Hinton Broth (MHB) until reaching 1 × 10^6^ CFU/mL for bacteria and 1 × 10^5^ CFU/mL for yeast. Peptide serial dilutions in dimethylsulfoxide (DMSO), ranging from 0.4 to 209.4 µM were prepared, and 2 µL of each peptide dilution was added to 198-µL diluted bacterial or yeast culture in a 96-well sterile plate (with five replicates). Sterile MHB and microbial culture with DMSO were negative controls. Plates were incubated for 16 h at 37°C. Microorganism growth was measured at 600 nm.

Hemolytic activity was determined using 200 µL of 4% red blood cell solution incubated with 200 µL of serial dilutions peptide (0.4–209.4 µM) in phosphate-buffered saline (PBS) 1× (five replicates). Negative controls contained PBS instead of peptide; positive controls contained 2% vol/vol Triton X-100 to yield complete hemolysis. Assays were incubated at 37°C for 2 h, and samples were centrifuged at 1,00 × *g* for 5 min. Two-hundred microliters of supernatant was transferred to a 96-well plate and absorbance was measured at 550 nm.

### Mammalian cell culture

RAW 264.7 murine macrophages and *Macaca mulatta* kidney cells (LLC-MK2) were cultured in 75-cm^2^ flasks with 10-mL Dulbecco’s Modified Eagle’s Medium (DMEM) supplemented with 10% fetal bovine serum and 1% penicillin/streptomycin (DMEM-10) medium. Culture conditions were 37°C, 5% CO_2_, and 95% relative humidity. RAW 264.7 cells were rinsed with PBS, detached with a cell scraper, and passaged every 96 h. LL-MCK2 were subcultured weekly, at 1:4 ratio, via trypsinization.

### Parasites

*Leishmania mexicana* strain M379, *P. falciparum* reference clones NF54 (chloroquine-sensitive, isolated from a patient at an airport in the Netherlands) and TM90C2B (C2B) (chloroquine, mefloquine, and atovaquone resistant, from Thailand) and *T. cruzi*, β-galactosidase-expressing, Tulahuen strain parasites (C4 clone; +lacZ, henceforth abbreviated as Tula β-gal) (33) were employed in the study.

### Parasite culture

*L. mexicana* promastigote stock cultures were maintained in USHMARU biphasic medium [blood agar slant overlayed with 3 mL of Schneider’s *Drosophila* medium (SDM), containing 10% fetal bovine serum (FBS)] at 25°C. Promastigotes were collected by centrifugation and washed with PBS, and culture medium was replaced every 2 days. Every 4 days, the parasites were transferred to a new tube and to a 25-cm^2^ tissue culture flask containing 10 mL of monophasic medium (SDM + 10% FBS + 1% penicillin/streptomycin). If present, rosettes were disrupted by passing the culture through 10-mL syringes with 27G needles.

*P. falciparum* was cultured in human O + erythrocytes in Roswell Park Memorial Institute medium 1640, supplemented with 25-mM HEPES buffer, 10-mM glucose, 2-mM glutamine, and O + human plasma. Parasites were cultured under low-oxygen atmosphere (5% O_2_, 5% CO_2_, and 90% N_2_) (34) and maintained in fresh human erythrocytes suspended at 4% hematocrit in complete medium at 37°C. Stock cultures were subpassaged every 3–4 days by transfer of infected red cells to a flask containing complete medium and uninfected erythrocytes.

*T. cruzi* trypomastigote culture was performed as reported previously (35, 36). LLC-MK2 cell monolayers were infected with 5 × 10^5^ trypomastigotes in 10-mL DMEM supplemented with 2% FBS and 1% penicillin/streptomycin (DMEM-2), for 48 h at 37°C, 5% CO_2_ and 98% relative humidity. Parasites were subsequently removed by rinsing with PBS, and 10-mL fresh DMEM-2 was added. Five days post-infection, trypomastigotes were harvested from the culture for the trypanocidal activity assays.

### *In vitro* anti-*Leishmania* activity assays

Parasite viability was measured colorimetrically via 3-(4,5-dimethylthiazol-2-yl)-2,5-diphenyltetrazole bromide tetrazolium salt (MTT) reduction. Promastigotes were placed in 96-well conical-bottom plates in SDM + 1% penicillin/streptomycin without FBS, at a density of 1 × 10^6^/well. Untreated parasites (growth control), 1-µM amphotericin B diluted in DMSO (positive control), 0.25% DMSO (negative control), and each of the peptides at a final concentration of 0.1, 0.5, 1.0, 5.0, and 10.0 µM were included in triplicate wells. Plates were incubated at 25°C for 48 h. Subsequently, 20-µL 10% MTT was added per well. Plates were incubated for 2 h in the dark at the same temperature and centrifuged at 4,000 rpm for 10 min, and the supernatant was removed. Formazan crystals were diluted with 50-µL DMSO, and absorbances (*A*_570–630_) were read (37, 38). Three independent assays were performed for each peptide.

### *In vitro* anti-*P*. *falciparum* activity assays

*In vitro* sensitivity to peptides was tested using a previously described SYBR green I fluorescence-based method (39, 40). Assays were set up in 96-well plates with twofold peptide dilutions in 150-μL total volume and 1.5% (vol/vol) final red blood cell concentration. Stock solutions of each peptide were prepared in DMSO. Experiments were started at an initial parasitemia of 0.5% (80% rings) synchronous parasite-infected red blood cells. Plates were incubated for 72 h at 37°C in an atmosphere of 5% CO_2_, 5% O_2_, and 90% N_2_. The SYBR green I dye-lysis mixture (l:100) was added to the parasites in black plates that were incubated at room temperature for an hour in the dark. The plates were then read using a fluorescence plate reader at excitation and emission wavelengths of 480 and 535 nm, respectively. Experiments were performed in duplicate wells. Three independent assays were performed for each peptide.

### Activity against *T. cruzi* trypomastigotes

Trypomastigotes were rinsed, suspended in phenol red-free and FBS-free Dulbecco’s Modified Eagle’s Medium, placed in 96-well plates (1 × 10^6^/well), and incubated with 100-µM chlorophenol-β-D-galactopyranoside red (CPRG) (41) in the presence of serial dilutions of AMPs (100.0- to 0.195-µM concentration range). Wells containing parasites not exposed to peptides and treated with 0.1% Triton served as 100% lysis reference. Plates were incubated in the dark at 37°C and 5% CO_2_ for 4 h, and absorbance was read at 590 nm. Experiments were performed in duplicate wells. Three independent assays were performed for each peptide.

### Activity against intracellular *T. cruzi* amastigotes

LLC-MK2 cells, 2 × 10^4^/well, were seeded and allowed to attach overnight to 96-well plates. he medium was removed and cells were infected with 1 × 10^5^ parasites in DMEM-2 for 24 h. Cell monolayers were washed four times with PBS, and serial dilutions of AMPs (100.0- to 0.195-µM range) in DMEM-2 without phenol red were added to duplicate wells. Wells containing infected cells not exposed to peptides served as parasite growth reference. The infection was allowed to proceed for 96 h. Subsequently, lysis solution was added to each well to a final concentration of 100-µM CPRG and 0.1% Triton and incubated for 2 h. Absorbance was measured at 590 nm (41). Three independent assays were performed for each peptide.

### Cytotoxicity over mammalian cells

The toxicity of the studied AMPs over LLC-MK2 and RAW 264 cells was determined via a resazurin (RZN) reduction assay (42). Briefly, 2 × 10^4^ cells/well were seeded in DMEM-10 in 96-well plates. Twenty-four hours later, culture medium was removed, and 10 twofold serial dilutions (100.0–0.195 µM) of each AMP in 200-µL volume were placed in duplicate wells. The plate was placed on the incubator in the dark at 37°C and 5% CO_2._ Ten microliters of 3-mM RZN sodium salt in PBS per well was added, and the plate was incubated for 24 h. Finally, the fluorescence was measured (530- to 560-nm excitation and 590-nm emission wavelengths). Three independent assays were performed for each peptide.

### Statistical data analysis

Half-maximal inhibitory concentration (IC_50_) and half-maximal cytotoxic concentration (CC_50_) with 95% confidence intervals in GraphPad Prism software (v.9.2.0., non-linear regression with curve fitting [model: log (inhibitor) vs response (three parameters)]. The selectivity index (SI) was determined by dividing the CC_50_ value of RAW 264.7 cells by the IC_50_ of the parasites.

### Peptide bioinformatics analysis

Sequence similarity between studied peptides and previous entries into the National Centre for Biotechnology Information database was explored with PSI-Blast (43, 44). Furthermore, studied peptide sequences were compared among each other using T-Coffee tool (45). Complete physicochemical and biochemical characterization for each peptide was performed using ExPasy (46), HeliQuest (47), and Peptide Mass Calculator (v.3.2). Peptide secondary structure was predicted using JPred (48), PSIPred (49), and SOPMA (50). With the information obtained from the prediction, the five peptides were modeled using Pymol and optimized using ChemBioDraw and Gaussian software. Optimized structures were employed for docking studies.

### Docking

Molecular docking simulations were performed using a phosphatidylcholine model containing 128 lipids and 2,460 water molecules simulated for 1.6 ns, which resemble eukaryotic cell membranes like those present in the studied parasites (51). Autodock tools were used to prepare the membrane model and peptide structures for calculations, which were performed using Autodock VINA (52), applying a 1-Å spacing, and a box size of 25 in *X*, 25 in *Y*, and 55 in *Z*. Exhaustiveness was set to 8 and full flexibility of the side chains was allowed.

## RESULTS

### Peptide synthesis and purity

Crude synthetic peptides presented a purity of 56% for CZS-1, 41% for CZS-4, 85% for CZS-16, 33% for DRS-SP2, and 42% for PTS-1. After purification, the five peptides were obtained in high purity (96%–98%), and their identities were confirmed via mass spectrum and corroborated with the theoretical mass (Table 1).

**TABLE 1:** Amino acid sequences and purity of the studied peptides

Peptide	Sequence	Theoretical mass (Da)	Monoisotopic mass MALDI-TOF MS^*a*^ (*m*/*z*)	Crude peptide (%)	Purified peptide (%)
CZS-1	GFLDIVKGVGKVALGAVSKLF-NH_2_	2,116.27	2,117.8	0.56	0.98
CZS-4	GFLDVIKHVGKAALSVVSHLINE-NH_2_	2,444.39	2,444.8	0.41	0.96
CZS-16	GFLDVLKGVGKAALGAVTHLINQ-NH_2_	2,319.34	2,320.6	0.85	0.98
DRS-SP2	ASWKVFLKNIGKAAGKAVLNSVTDMVNQ-NH_2_	2,987.63	2,988.3	0.33	0.98
PTS-1	GFLDTLKNIGKTVGRIALNVLT-NH_2_	2,341.38	2,342.2	42	0.98

^*a*^ MALDI-TOF MS, matrix-assisted laser desorption/ionization time-of-flight mass spectrometry.

### Peptide bioinformatic characterization

**Fig 1:**
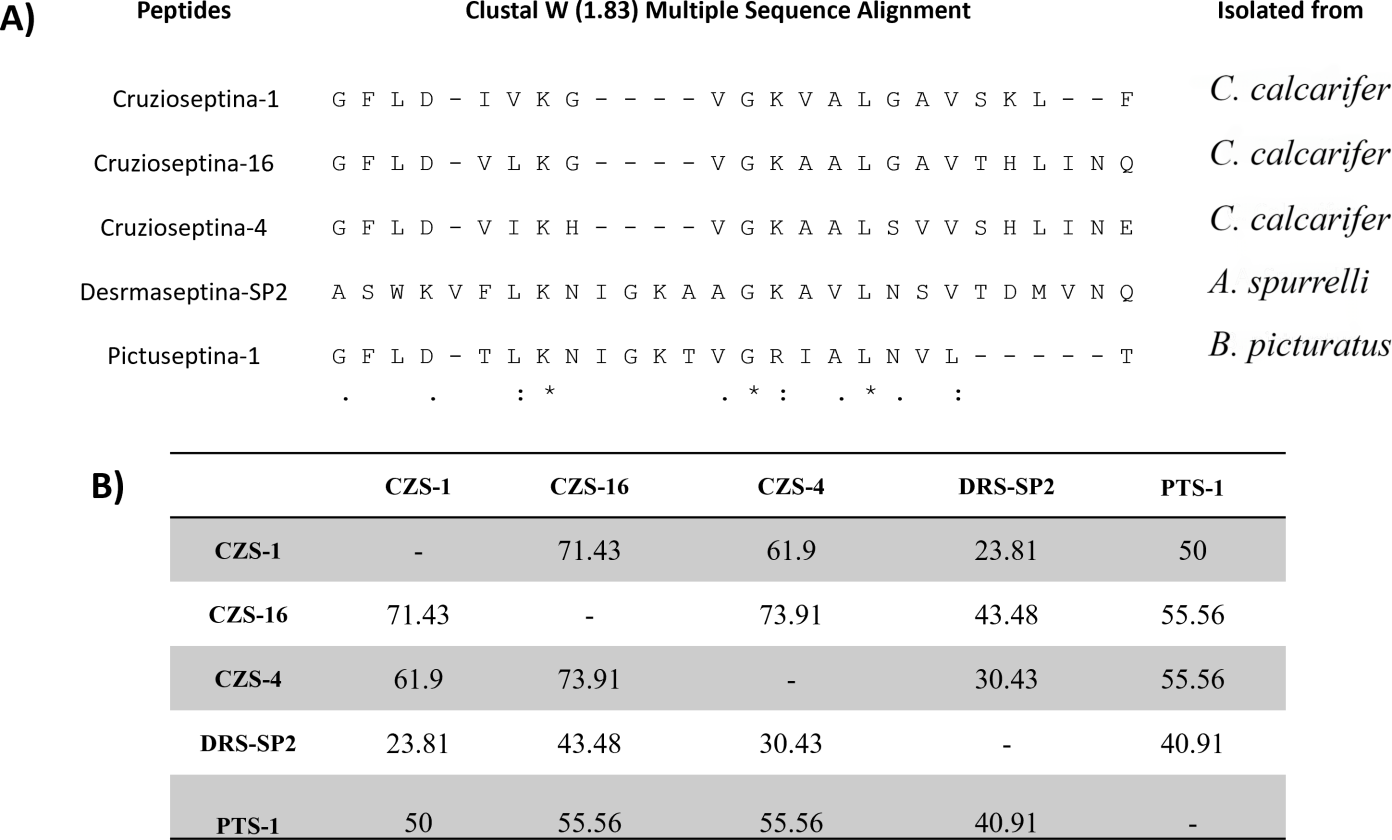
Multiple sequence alignment (A) and identity matrix (B) of the studied peptides. Three conserved residues (glycine, leucine, and lysine) are present in all studied peptides. Glycine and leucine are hydrophobic amino acids, while lysine is basic and contributes to the positive charge of the peptides. The physicochemical characterization of the peptides is depicted in Table 2. Peptides are composed of 300–400 atoms and 21–28 amino acids, corresponding to molecular weights between 2,118 and 2,990 Da. All of them are basic and positively charged at physiological pH. Their isoelectric point is higher than 7, and the number of their basic residues doubles that of negative ones. Hydrophobic amino acids constitute around 50% of the structure of each peptide.

A comparison of the amino acid sequences of the five peptides is shown in Fig. 1. CZS-1, CZS-4, and CZS-16 belong to the same peptide family and display high similarity (>61.90%). DRS-SP2 yielded high identity with other dermaseptins, including DRS-SP1, DRS-TR1, DRS-PS2, and DRS-DI2 (identity percentage 84.62%–92.31%). The highest identity percentage found between the cruzioseptin family and DRS-SP2 was with CZS-16 (43.48%). Regarding PTS-1, this peptide showed >50% similarity with cruzioseptin members, the highest with CZS-4 and CZS-16 (55.56%).

**TABLE 2:** Physicochemical characterization of the five studied peptides

Parameter	CZS-1	CZS-4	CZS-16	DRS-SP2	PTS-1
No. of amino acids (aa)	21	23	23	28	25
Molecular weight	2,118.59	2,429.89	2,321.75	2,990.51	2,581.10
pI	9.70	8.61	8.60	10.80	9.83
Formula	C_101_H_168_N_24_O_25_	C_112_H_185_N_31_O_29_	C_106_H_177_N_29_O_29_	C_134_H_221_N_37_O_38_S_1_	C_118_H_202_N_32_O_32_
Atom number	318	357	341	431	384
Neg. aa	1	1	1	1	2
Pos. aa	3	2	2	4	5
C-terminal	-NH_2_	-NH_2_	-NH_2_	-NH_2_	-NH_2_
Net charge pH 7	4.00	3.22	3.11	4.00	3.00
Hydrophobicity (H)	0.581	0.530	0.449	0.358	0.489
% neutral aa	23.81	26.09	30.43	32.14	20.00
% basic aa	14.29	18.39	13.04	14.29	20.00
% acid aa	4.76	4.35	4.35	3.57	8.00
% hydrophobic aa	57.14	52.17	52.17	50.00	52.00

### Anti-microbial and hemolytic activity of CZS-4

**Fig 2:**
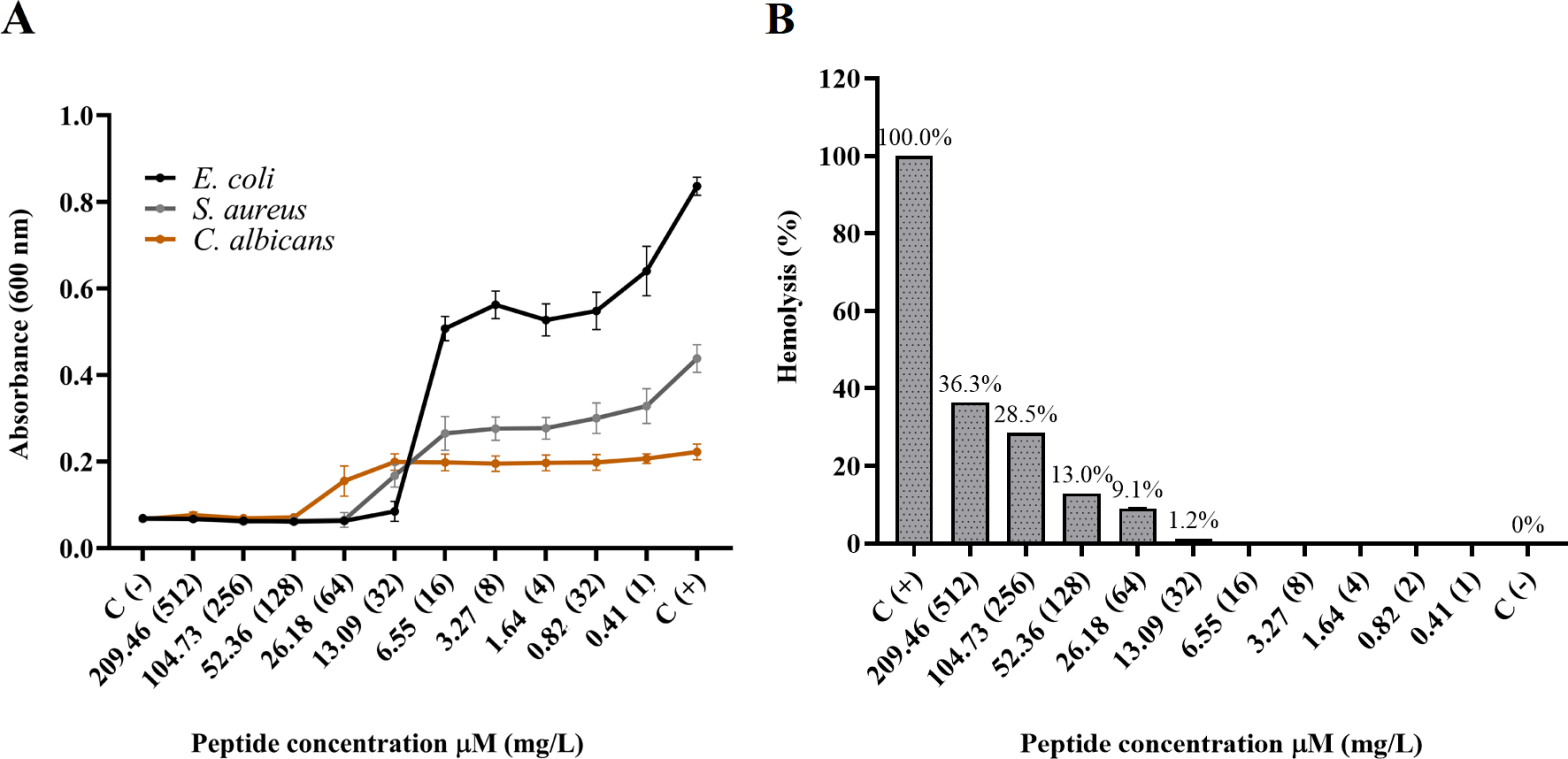
Anti-microbial and hemolytic activities of the CZS-4 peptide. (**A**) Inhibitory effect of CZS-4 against *E. coli*, *S. aureus*, and *C. albicans*. (**B**) Hemolysis caused by CZS-4. One hundred percent of hemolysis was determined using Triton X-100.

Except for CZS-4, we have previously reported on the activity of all peptides included in this study against bacteria and *Candida albicans*, as well as their hemolytic activity (28–31). Here, we show that CZS-4 displayed anti-microbial activity against bacteria (*E. coli* and *S. aureus*) and yeast (*Candida albicans*). The lowest MIC value obtained was against *E. coli* (13.09 µM) followed by *S. aureus* (26.18 µM). The highest MIC corresponded to the yeast *C. albicans* with a value of 52.36 µM (Fig. 2A). On the other hand, CZS-4 induced hemolysis of 13% at 128 µM. While in anti-bacterial concentration, this peptide showed hemolysis of less than 9.1% (Fig. 2B).

### Cytotoxicity of peptides over mammalian cells

**Fig 3:**
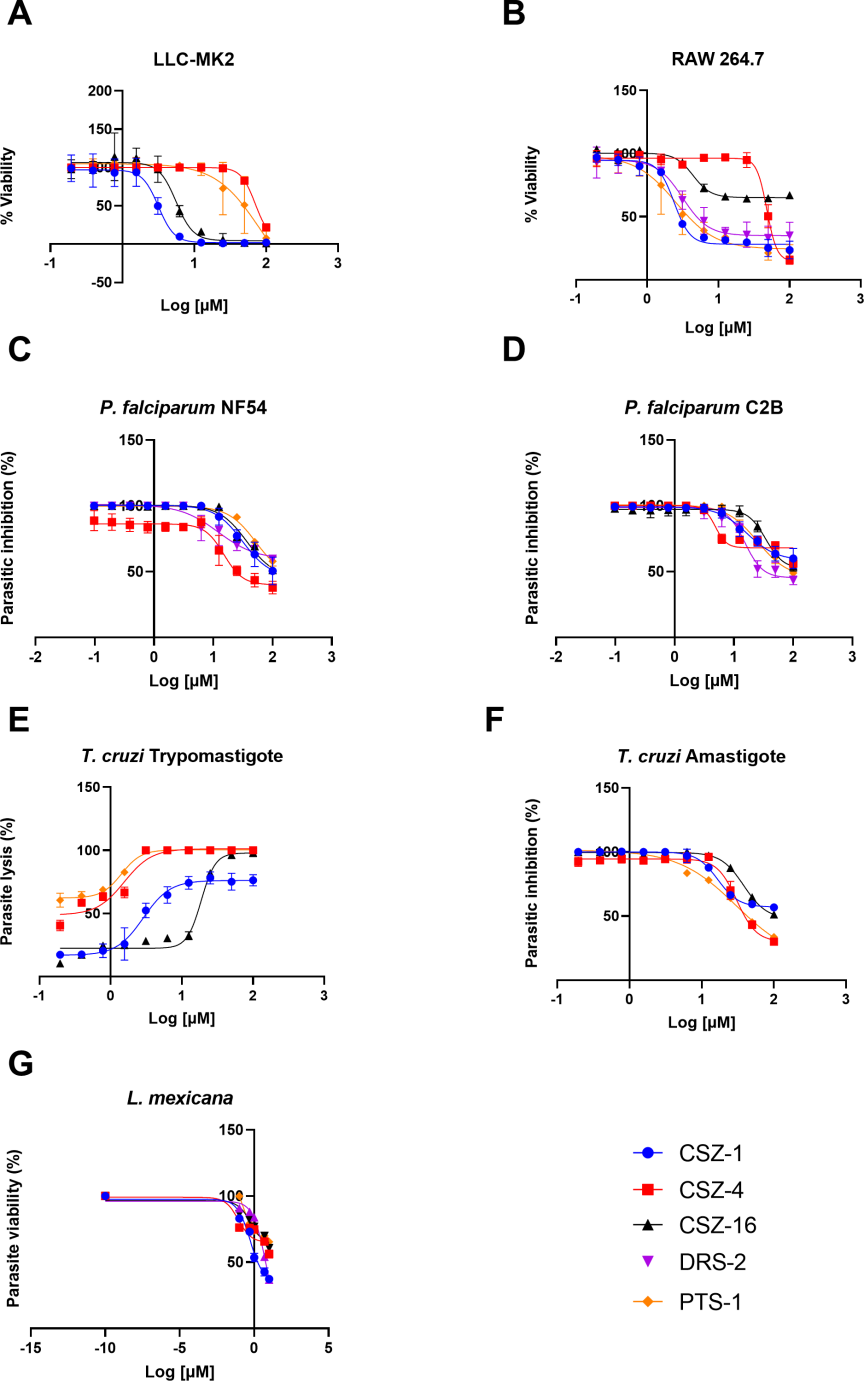
Dose-response curves for the activity of the studied synthetic peptides against mammalian cells (**A and B**) and protozoan parasites (**C–G**). Data are shown as mean values of independent assays ± standard deviation.

All studied AMPs displayed some degree of cytotoxicity against mammalian cells (Table 3; Fig. 3A and B). CZS-1 displayed IC_50_ values of 3.17 and 2.38 µM against LL-MCK2 and RAW macrophages, respectively. Interestingly, CZS-4 caused very low cytotoxicity, with IC_50_ values of 70.6 µM for LL-MCK-2 cells and 47.96 µM for RAW macrophages. Meanwhile, CZS-16 and DRS-2 displayed cytotoxic activity against both types of mammalian cells, ranging from 3.14 to 5.59 µM. In a case where mammalian cell types were affected very differently, PST-1 displayed an IC_50_ of 80.07 µM against LL-MCK-2 cells and a much lower level against RAW macrophages (IC_50_ = 2.52 µM).

**TABLE 3:** Anti-parasitic activity and cytotoxicity of AMPs^*a*^

Peptide	Llc-mk2	Raw 264.7	*T. cruzi*		*P. falciparum*		*L. mexicana*
			Trypomastigotes	Amastigotes	NF54	TM90C2B	
CZS-1	3.17 (2.70–3.75)	2.38 (2.13–2.65)	2.87 (2.35–3.53)	16.72 (15.56–18.00)	31.16 (23.21–57.99)	16.76 (12.88–24.45)	0.54 (0.43–0.68)
CZS-4	70.66 (NA-90.46)	47.96 (44.93–51.12)	1.55 (0.65–2.22)	30.65 (26.88–35.57)	13.86 (10.26–22.52)	4.87 (3.87–5.85)	0.09 (0.04–0.25)
CZS-16	5.59 (4.64–6.86)	4.40 (3.90–4.95)	18.7 (16.56–20.98)	38.33 (35.44–41.97)	36.82 (27.69–61.83)	34.41 (29.16–42.70)	6.46 (4.87–8,83)
DRS-SP2	ND	3.14 (2.45–4.10)	ND	ND	12.06 (7.57–32.84)	15.34 (13.24–18.00)	0.61 (0.37–0.98)
PTS-1	80.07 (30.49-NA)	2.52 (1.57–3.79)	1.42 (1.25–1.58)	30.16 (22.93–47.83)	52.56 (41.46–96.79)	24.87 (20.43–32.89)	0.10 (0.07–0.14)

^*a*^ Data are reported as IC_50_ values (µM) and 95% confidence intervals. ND, not done.

### Anti-parasitic activity of studied AMPs

#### L. mexicana

Promastigotes displayed the greatest AMP sensitivity among the studied parasites. Promastigotes were incubated with AMPs for 48 h at different concentrations. *L. mexicana* was susceptible to all studied peptides, with IC_50_ values ranging from 0.09 to 10.0 µM (Table 3). CZS-4 appeared to be the most effective of all the tested AMPs, while CZS-16 showed a reduced effect.

#### P. falciparum

The studied peptides had anti-plasmodial activity against the drug-sensitive NF54 and the multiple drug-resistant C2B strains of *P. falciparum*. IC_50_ values ranged from 4.87 to 52.56 µM, where DRS-SP2 and CZS-4 showed higher activity in both strains (Table 3).

#### T. cruzi

CZS-1, CZS-4 and PTS-1 displayed potent activity against *T. cruz*i trypomastigotes, with IC_50_ values between 1.42–2.87 µM (Table 3). CZS-16 had a noticeably larger IC_50_ value (18.70 µM). Conversely, much higher IC_50_ values (16.72–38.33 µM) were recorded against intracellular amastigotes.

### SI

As depicted in Table 4, CZS-4 showed the highest SI against *L. mexicana* and *T. cruzi* trypomastigotes at 532.89 and 30.94, respectively. On the other hand, CZS-16 is selectively active against mammalian cells. PST-1 also displayed low selectivity against *T. cruzi* and *P. falciparum*, with SI values from 0.05 and 1.775. PST-1’s selectivity for *L. mexicana* was much greater, with an SI of 25.20.

**TABLE 4:** SI of studied AMPs^*a*^^,*b*^

Peptide	*T. cruzi*		*P. falciparum*		*L. mexicana*
	Trypomastigotes	Amastigotes	NF54	C2B	
CZS-1	0.83	0.14	0.08	0.14	4.41
CZS-4	30.94	1.56	3.46	9.85	532.89
CZS-16	0.24	0.11	0.12	0.13	0.44
DRS-SP2	ND	ND	0.26	0.20	5.15
PTS-1	1.775	0.08	0.05	0.10	25.20

^*a*^ SI denotes RAW 264.7 CC_50_/parasite IC_50_.

^*b*^ Larger values indicate greater peptide specificity for parasites over host cells.

### Structural model prediction

**Fig 4:**
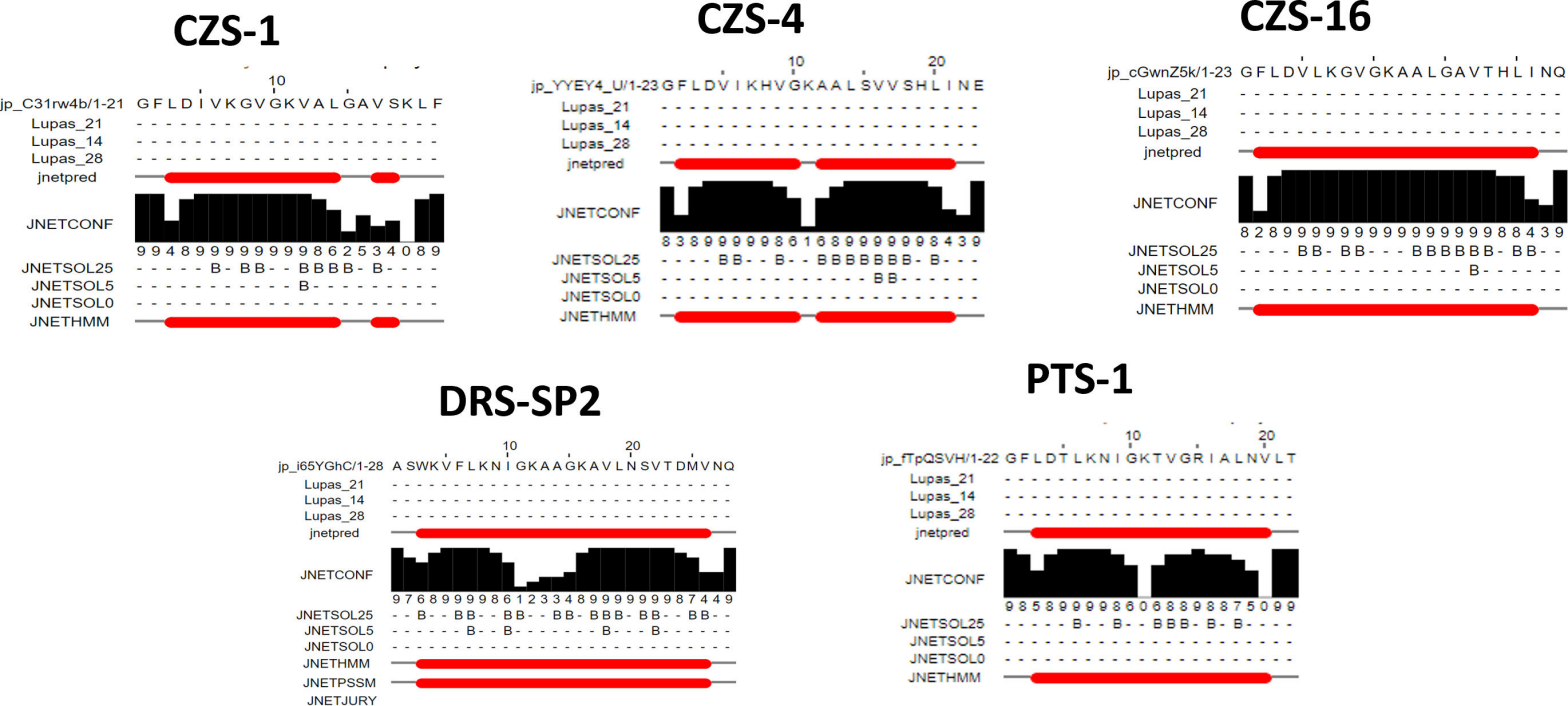
Secondary structure prediction for the five studied peptides obtained with JPred, PSIPred, and SOPMA bioinformatic tools.

**Fig 5:**
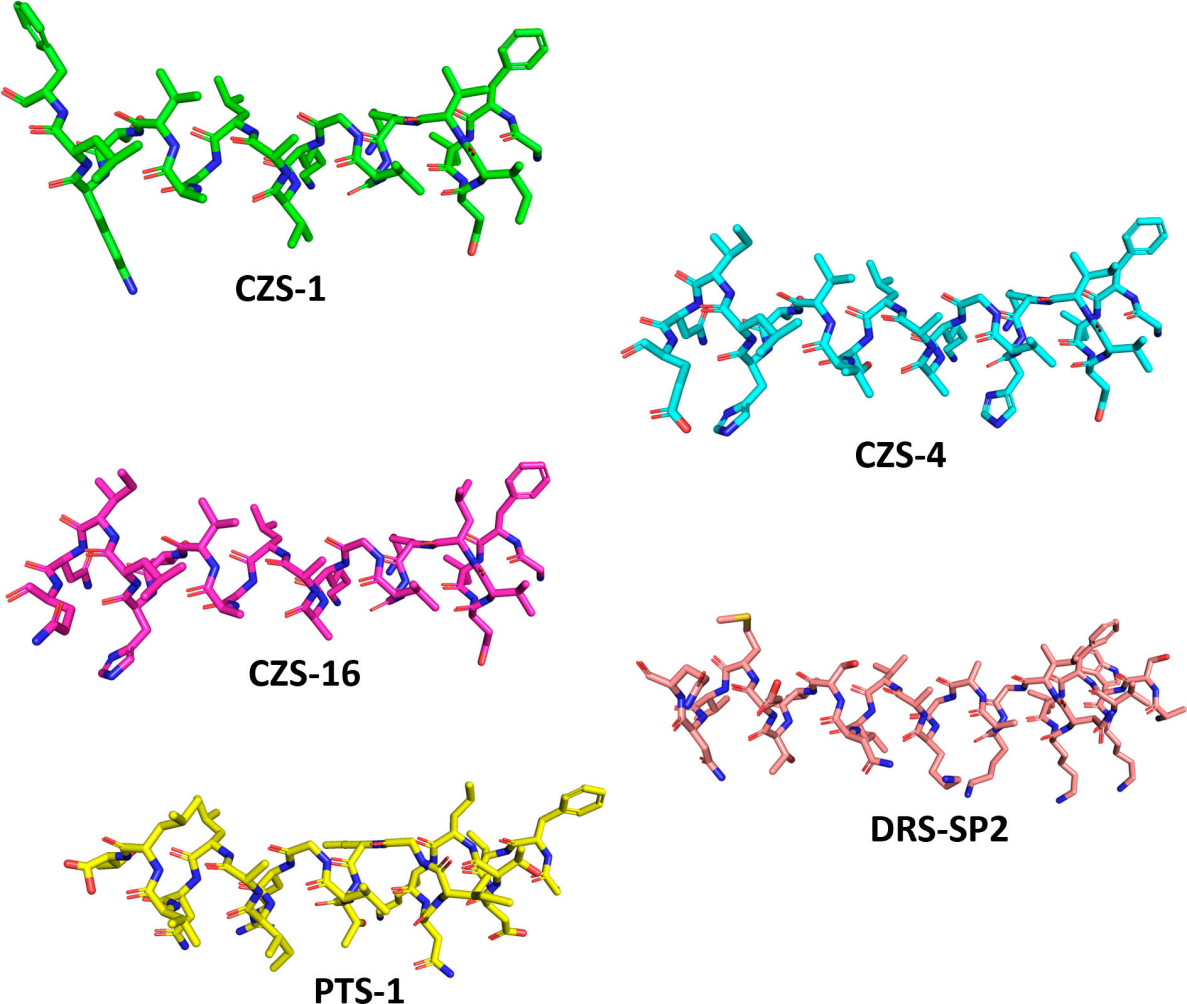
Optimized three-dimensional structure of the studied peptides obtained using ChemBioDraw and Gaussian software.

Secondary structure analysis predicts an alpha-helical conformation for all peptides (Fig. 4), in agreement with the secondary structures reported in the literature for other members of their families and for other anti-microbial peptides found in different species of frogs. The five peptides were modeled in an alpha-helical secondary structure and optimized. Optimized three-dimensional peptide structures are shown in Fig. 5.

### Molecular docking

**Fig 6:**
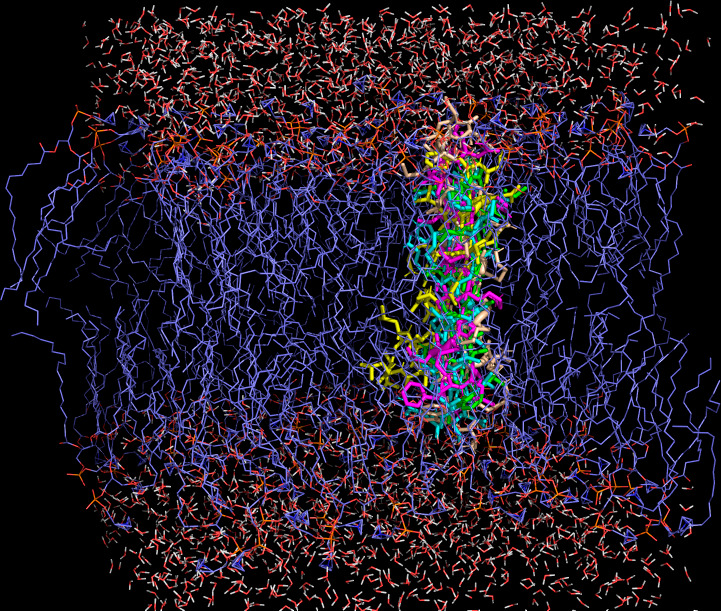
Docking of studied AMPs in a model of the parasite’s cellular membrane. Peptide colors are the same as those in Fig. 5.

A membrane model containing 128 phosphatidylcholine molecules and 2,460 water molecules was chosen because it adequately represents eukaryotic pathogen cell membranes. Our analysis predicts favorable interaction between this membrane model and optimized peptide structures, as indicated by the negative docking scores obtained, which ranged from −5.4 kcal/mol in CZS-16 to −7.6 kcal/mol in CZS-1. Additionally, the most favorable interaction conformation for all studied peptides would be to locate along the lipid bilayer (Fig. 6), which would in turn be predicted to cause membrane destabilization, ultimately resulting in parasite lysis.

## DISCUSSION

Amphibian skin AMPs have been extensively studied and proposed as therapeutic alternatives for the treatment of infectious diseases, including those caused by multidrug-resistant microorganisms. To date, more than 1,000 amphibian AMPs with broad structural and biological diversity have been reported (53). Anti-parasitic activity has been described in the dermaseptin peptide family, but the recently described cruzioseptin and pictuseptin peptide families are anti-bacterial and anti-fungal peptides with unexplored anti-parasitic activity (28, 30, 31).

In this study, we report findings on the bioactivity of CZS-4 against bacteria and yeast, and CZS-1, CZS-4, CZS-16, DRS-SP2, and PTS-1 against mammalian cells and protozoan parasites. CZS-4 exhibited anti-microbial activity against *S. aureus*, *C. albicans*, and *E. coli*, similar to what was previously reported for other cruzioseptin family members (29, 30). Interestingly, *C. albicans* is much less sensitive to the peptide compared to the bacteria, likely because of the resistance conferred by the fungal cell wall structure. MICs differ among cruzioseptins, with CZS-1 being the most potent one. It has been proposed that the biological activity of AMPs is correlated with certain physicochemical characteristics, such as hydrophobicity, high net charge, and helicity (20). Therefore, an exhaustive study must be performed to determine the physicochemical properties and structural determinants required to induce a potent effect against Gram-negative bacteria, Gram-positive bacteria, and yeasts.

The five studied peptides (CZS-1, CZS-4, CZS-16, DRS-SP2, and PTS-1) display anti-microbial properties, as shown here for CZS-4 and in previous reports for the other peptides (28–31). Additionally, we show here that these peptides also possess varying degrees of activity against the protozoan parasites *L. mexicana*, *P. falciparum*, and *T. cruzi*.

As expected, when the studied AMPs were tested against the mammalian-specific life cycle stages of *T. cruzi*, they displayed greater activity against trypomastigotes than intracellular amastigotes. These findings were similar to those from other AMPs with trypanocidal activity, including melittin (54), NK-lysin (55), and several dermaseptins (24, 56). Conversely, the effect on intracellular amastigotes is probably reduced because peptides must first cross the host cell’s plasma membrane in order to reach the cytoplasm, where amastigotes multiply (55). Persistence of intracellular amastigote nests in different host tissues, including cardiac tissue, is crucial for long-term parasite survival during chronic Chagas disease (57). In this sense, the elimination of amastigotes is crucial, and therefore, peptide intracellular delivery and associated cytotoxicity are important challenges to be overcome for a peptide-based treatment.

While there was anti-malarial activity against the erythrocytic stages of both resistant and sensitive strains of *P. falciparum*, this activity was generally modest for most peptides and lower than some previously related peptides such as dermaseptin S4 derivatives (23). DRS-SP2 and CZS-4 had the highest activity against *P. falciparum*, which was comparable to derivatives of DRS S3 and DRS S4 (22), suggesting that further research of these peptides can result in more promising activities. To reach the intracellular malaria parasite membrane, the peptides have to cross the erythrocytic membrane and enter the parasitophorous membrane, which may explain the lower activity when compared to other parasites (55).

As for *L. mexicana*, CZS-1, CZS-4, CZS-16, DRS-SP2, and PTS-1 demonstrated great leishmanicidal activity against promastigotes. These peptides were more active compared to other anti-microbial peptides isolated from amphibians (58) and marine sources (59). Although *L. mexicana* promastigotes were more sensitive to these AMPs than *T. cruzi* trypomastigotes, a limitation of our study is the lack of data for intracellular *Leishmania* amastigotes. Testing the studied peptides against both intracellular and extracellular forms of *T. cruzi* was possible because of the availability of the recombinant, beta-galactosidase expressing strain (Tula-beta gal). However, the colorimetric method employed to quantify the viability of *L. mexicana* parasites (MTT reduction) is not useful with intracellular parasites because the signal produced by the parasites is indistinguishable from that caused by the host cell, confounding the results. Therefore, a direct comparison of the sensitivity of the (clinically relevant) intracellular amastigotes of both parasite species is not possible from our data. Likely, intracellular amastigotes of *L. mexicana* will be less sensitive to the peptides, as is the case for *T. cruzi*.

Most AMPs interact with the plasma membrane of microorganisms as an initial mechanism of action. Several members of the cruzioseptin and dermaseptin families have shown a membranolytic effect on *S. aureus*, *T. cruzi*, and *Leishmania* spp. (32, 56, 60). Based on this observation and on our research, we believe that CZS-1, CZS-4, CZS-16, DRS-SP2, and PTS-1 act on parasites via membrane destabilization. Our hypothesis is supported by the results of molecular docking, which indicate a peptide-membrane interaction as evidenced by negative docking values. Previous computational studies have also shown that there is a favorable interaction between anti-microbial peptides and phospholipid bilayers (61, 62).

Toxicity is an important concern when studying peptides for drug development (63). In our study, this challenge was evaluated exposing murine macrophages and LLC-MK2 cells to the peptides. All studied peptides displayed activity against RAW 264.7 macrophages; however, the activity was quite low for CZS-4. These data are relevant for anti-parasitic drugs, given that macrophages are one of the cells that parasites invade in the vertebrate host. Indeed, CZS-4 has great specificity for targeting *L. mexicana* promastigotes (SI = 532.89) and *T. cruzi* trypomastigotes (SI = 30.94). Conversely, CZS-4 and PTS-1 induced low cytotoxicity toward LL-MCK_2_ cells. (CC_50_ = 70.66 and 80.07 µM, respectively), while CZS-1 and CZS-16 were highly cytotoxic (CC_50_ = 3.17 and 5,59 µM, respectively). Currently, several strategies have been used with the aim of reducing bioactive peptide toxicity, including cyclization, incorporation of D-amino acids, peptides, and computational techniques (63).

Although *in vitro* studies are a useful starting point for characterization of AMP biological properties, their anti-microbial and anti-parasitic activities as well as cytotoxicity may differ greatly *in vivo*. Therefore, additional studies are warranted to clarify the therapeutic potential of the studied peptides.

In conclusion, we have shown that five amphibian AMPs, with anti-bacterial and anti-fungal properties previously reported, also display differing degrees of activity against the protozoan parasites *L. mexicana*, *P. falciparum,* and *T. cruzi* at micromolar concentrations. As expected, peptides were more active against extracellular parasite forms. Among the studied peptides, CZS-4 is the most promising due to its low toxicity and high efficacy in eliminating parasites in *in vitro* assays. In addition, bioinformatic analysis suggest that these peptides act through a membranolytic effect.

## References

[1] Lidani KCF, Andrade FA, Bavia L, Damasceno FS, Beltrame MH, Messias-Reason IJ, Sandri TL. 2019. Chagas disease: from discovery to a worldwide health problem. Front Public Health 7:166. doi:10.3389/fpubh.2019.00166 31312626 PMC6614205

[2] Burza S, Croft SL, Boelaert M. 2018. Leishmaniasis. Lancet 392:951–970. doi:10.1016/S0140-6736(18)31204-2 30126638

[3] Recht J, Siqueira AM, Monteiro WM, Herrera SM, Herrera S, Lacerda MVG. 2017. Malaria in Brazil, Colombia, Peru and Venezuela: current challenges in malaria control and elimination. Malar J 16:161. doi:10.1186/s12936-017-1925-6 28676055 PMC5496604

[4] Mansoldo FRP, Carta F, Angeli A, Cardoso V da S, Supuran CT, Vermelho AB. 2020. Chagas disease: perspectives on the past and present and challenges in drug discovery. Molecules 25:5483. doi:10.3390/molecules25225483 33238613 PMC7700143

[5] Zulfiqar B, Shelper TB, Avery VM. 2017. Leishmaniasis drug discovery: recent progress and challenges in assay development. Drug Discov Today 22:1516–1531. doi:10.1016/j.drudis.2017.06.004 28647378

[6] Miles MA, Feliciangeli MD, de Arias AR. 2003. American trypanosomiasis (Chagas’ disease) and the role of molecular epidemiology in guiding control strategies. BMJ 326:1444–1448. doi:10.1136/bmj.326.7404.1444 12829559 PMC1126319

[7] Chagas disease (also known as American Trypanosomiasis). Available from: https://www.who.int/news-room/fact-sheets/detail/chagas-disease-(american-trypanosomiasis). Retrieved 06 Sep 2022.

[8] Moncayo A, Silveira AC. 2017. Current epidemiological trends of chagas disease in Latin America and future challenges: epidemiology, surveillance, and health policies, p 59–88. In Am Trypanos Chagas DIS one hundred years RES second Ed10.1590/s0074-02762009000900005 19753454

[9] Henao-Martínez AF, Schwartz DA, Yang IV. 2012. Chagasic cardiomyopathy, from acute to chronic: is this mediated by host susceptibility factors? Trans R Soc Trop Med Hyg 106:521–527. doi:10.1016/j.trstmh.2012.06.006 22819769

[10] Mazzeti AL, Gonçalves KR, Mota SLA, Pereira DE, Diniz L de F, Bahia MT. 2021. Combination therapy using nitro compounds improves the efficacy of experimental Chagas disease treatment. Parasitology 148:1320–1327. doi:10.1017/S0031182021001001 34247670 PMC11010181

[11] Sales Junior PA, Molina I, Fonseca Murta SM, Sánchez-Montalvá A, Salvador F, Corrêa-Oliveira R, Carneiro CM. 2017. Experimental and clinical treatment of Chagas disease: a review. Am J Trop Med Hyg 97:1289–1303. doi:10.4269/ajtmh.16-0761 29016289 PMC5817734

[12] Torres-Guerrero E, Quintanilla-Cedillo MR, Ruiz-Esmenjaud J, Arenas R. 2017. Leishmaniasis: a review. F1000Res 6:750. doi:10.12688/f1000research.11120.1 28649370 PMC5464238

[13] Tabbabi A. 2019. Review of leishmaniasis in the middle East and North Africa. Afr Health Sci 19:1329–1337. doi:10.4314/ahs.v19i1.4 31148958 PMC6531937

[14] Fontecha G, Sánchez A, Ortiz B. 2021. Publication trends in neglected tropical diseases of Latin America and the Caribbean: a bibliometric analysis. Pathogens 10:356. doi:10.3390/pathogens10030356 33802834 PMC8002643

[15] Yurchenko V, Chistyakov DS, Akhmadishina LV, Lukashev AN, Sádlová J, Strelkova MV. 2023. Revisiting epidemiology of leishmaniasis in central Asia: lessons learnt. Parasitology 150:129–136. doi:10.1017/S0031182022001640 36453145 PMC10090592

[16] Greenwood BM, Bojang K, Whitty CJM, Targett GAT. 2005. Malaria. Lancet 365:1487–1498. doi:10.1016/S0140-6736(05)66420-3 15850634

[17] Bergaoui I, Zairi A, Tangy F, Aouni M, Selmi B, Hani K. 2013. In vitro antiviral activity of dermaseptin S(4) and derivatives from amphibian skin against herpes simplex virus type 2. J Med Virol 85:272–281. doi:10.1002/jmv.23450 23161023

[18] El-Dirany R, Shahrour H, Dirany Z, Abdel-Sater F, Gonzalez-Gaitano G, Brandenburg K, Martinez de Tejada G, Nguewa PA. 2021. Activity of anti-microbial peptides (AMPs) against Leishmania and other parasites: an overview. Biomolecules 11:984. doi:10.3390/biom11070984 34356608 PMC8301979

[19] Navon-Venezia S, Feder R, Gaidukov L, Carmeli Y, Mor A. 2002. Antibacterial properties of dermaseptin S4 derivatives with in vivo activity. Antimicrob Agents Chemother 46:689–694. doi:10.1128/AAC.46.3.689-694.2002 11850249 PMC127478

[20] Santana CJC, Magalhães ACM, Dos Santos Júnior ACM, Ricart CAO, Lima BD, Álvares A da CM, Freitas SM de, Pires OR, Fontes W, Castro MS. 2020. Figainin 1, a novel amphibian skin peptide with antimicrobial and antiproliferative properties. Antibiotics (Basel) 9:625. doi:10.3390/antibiotics9090625 32967114 PMC7559428

[21] Strahilevitz J, Mor A, Nicolas P, Shai Y. 1994. Spectrum of antimicrobial activity and assembly of dermaseptin-b and its precursor form in phospholipid membranes. Biochemistry 33:10951–10960. doi:10.1021/bi00202a014 8086412

[22] Efron L, Dagan A, Gaidukov L, Ginsburg H, Mor A. 2002. Direct interaction of dermaseptin S4 aminoheptanoyl derivative with intraerythrocytic malaria parasite leading to increased specific antiparasitic activity in culture. J Biol Chem 277:24067–24072. doi:10.1074/jbc.M202089200 11937508

[23] Krugliak M, Feder R, Zolotarev VY, Gaidukov L, Dagan A, Ginsburg H, Mor A. 2000. Antimalarial activities of dermaseptin S4 derivatives. Antimicrob Agents Chemother 44:2442–2451. doi:10.1128/AAC.44.9.2442-2451.2000 10952593 PMC90083

[24] Brand GD, Leite J, Silva LP, Albuquerque S, Prates MV, Azevedo RB, Carregaro V, Silva JS, Sá VCL, Brandão RA, Bloch C. 2002. Dermaseptins from Phyllomedusa oreades and Phyllomedusa distincta. Anti-Trypanosoma cruzi activity without cytotoxicity to mammalian cells. J Biol Chem 277:49332–49340. doi:10.1074/jbc.M209289200 12379643

[25] Brand G.D, Santos RC, Arake LM, Silva VG, Veras LMC, Costa V, Costa CHN, Kuckelhaus SS, Alexandre JG, Feio MJ, Leite JRSA. 2013. The skin secretion of the amphibian Phyllomedusa nordestina: a source of antimicrobial and antiprotozoal peptides. Molecules 18:7058–7070. doi:10.3390/molecules18067058 23774944 PMC6270157

[26] Zampa MF, Araújo IMS, Costa V, Nery Costa CH, Santos JR, Zucolotto V, Eiras C, Leite J. 2009. Leishmanicidal activity and immobilization of dermaseptin 01 antimicrobial peptides in ultrathin films for nanomedicine applications. Nanomedicine 5:352–358. doi:10.1016/j.nano.2008.11.001 19215729

[27] Ghosh JK, Shaool D, Guillaud P, Cicéron L, Mazier D, Kustanovich I, Shai Y, Mor A. 1997. Selective cytotoxicity of dermaseptin S3 toward intraerythrocytic Plasmodium falciparum and the underlying molecular basis. J Biol Chem 272:31609–31616. doi:10.1074/jbc.272.50.31609 9395500

[28] Morán-Marcillo G, Sánchez Hinojosa V, de los Monteros-Silva NE, Blasco-Zúñiga A, Rivera M, Naranjo RE, Almeida JR, Wang L, Zhou M, Chen T, Shaw C, Proaño-Bolaños C. 2022. Picturins and Pictuseptins, two novel antimicrobial peptide families from the skin secretions of the Chachi treefrog, Boana picturata. J Proteomics 264:104633. doi:10.1016/j.jprot.2022.104633 35640793

[29] Cuesta SA, Reinoso C, Morales F, Pilaquinga F, Morán-Marcillo G, Proaño-Bolaños C, Blasco-Zúñiga A, Rivera M, Meneses L. 2021. Novel antimicrobial cruzioseptin peptides extracted from the splendid leaf frog, Cruziohyla calcarifer. Amino Acids 53:853–868. doi:10.1007/s00726-021-02986-w 33942149

[30] Proaño-Bolaños C, Zhou M, Wang L, Coloma LA, Chen T, Shaw C. 2016. Peptidomic approach identifies cruzioseptins, a new family of potent antimicrobial peptides in the splendid leaf frog, Cruziohyla calcarifer. J Proteomics 146:1–13. doi:10.1016/j.jprot.2016.06.017 27321580

[31] Proaño-Bolaños C, Blasco-Zúñiga A, Almeida JR, Wang L, Llumiquinga MA, Rivera M, Zhou M, Chen T, Shaw C. 2019. Unravelling the skin secretion peptides of the gliding leaf frog, Agalychnis spurrelli (Hylidae). Biomolecules 9:667. doi:10.3390/biom9110667 31671555 PMC6920962

[32] Mendes B, Proaño-Bolaños C, Gadelha FR, Almeida JR, Miguel DC. 2020. Cruzioseptins, antibacterial peptides from Cruziohyla calcarifer skin, as promising leishmanicidal agents. Pathog Dis 78:ftaa053. doi:10.1093/femspd/ftaa053 32926094

[33] Buckner FS, Verlinde CL, La Flamme AC, Van Voorhis WC. 1996. Efficient technique for screening drugs for activity against Trypanosoma cruzi using parasites expressing beta-galactosidase. Antimicrob Agents Chemother 40:2592–2597. doi:10.1128/AAC.40.11.2592 8913471 PMC163582

[34] Trager W, Jensen JB. 1976. Human malaria parasites in continuous culture. Science 193:673–675. doi:10.1126/science.781840 781840

[35] Andrews NW, Alves MJM, Schumacher RI, Colli W. 1985. Trypanosoma cruzi: protection in mice immunized with 8-methoxypsoralen-inactivated trypomastigotes. Exp Parasitol 60:255–262. doi:10.1016/0014-4894(85)90029-3 3935472

[36] Caler EV, De Avalos SV, Haynes PA, Andrews NW, Burleigh BA. 1998. Oligopeptidase B-dependent signaling mediates host cell invasion by Trypanosoma cruzi. EMBO J 17:4975–4986. doi:10.1093/emboj/17.17.4975 9724634 PMC1170826

[37] Veiga A, Albuquerque K, Corrêa ME, Brigido H, Silva e Silva J, Campos M, Silveira F, Santos L, Dolabela M. 2017. Leishmania amazonensis and Leishmania chagasi: in vitro leishmanicide activity of Virola surinamensis (rol.) warb. Exp Parasitol 175:68–73. doi:10.1016/j.exppara.2017.02.005 28174103

[38] Martín-Quintal Z, Moo-Puc R, González-Salazar F, Chan-Bacab MJ, Torres-Tapia LW, Peraza-Sánchez SR. 2009. In vitro activity of Tridax procumbens against promastigotes of Leishmania mexicana. J Ethnopharmacol 122:463–467. doi:10.1016/j.jep.2009.01.037 19429313

[39] Kyle DE, Oduola AMJ, Martin SK, Milhous WK. 1990. Plasmodium falciparum: modulation by calcium antagonists of resistance to chloroquine, desethylchloroquine, quinine, and quinidine in vitro. Trans R Soc Trop Med Hyg 84:474–478. doi:10.1016/0035-9203(90)90004-x 2091331

[40] Smilkstein M, Sriwilaijaroen N, Kelly JX, Wilairat P, Riscoe M. 2004. Simple and inexpensive fluorescence-based technique for high-throughput antimalarial drug screening. Antimicrob Agents Chemother 48:1803–1806. doi:10.1128/AAC.48.5.1803-1806.2004 15105138 PMC400546

[41] Bettiol E, Samanovic M, Murkin AS, Raper J, Buckner F, Rodriguez A. 2009. Identification of three classes of heteroaromatic compounds with activity against intracellular Trypanosoma cruzi by chemical library screening. PLoS Negl Trop Dis 3:e384. doi:10.1371/journal.pntd.0000384 19238193 PMC2639639

[42] Kumar P, Nagarajan A, Uchil PD. 2018. Analysis of cell viability by the alamarBlue assay. Cold Spring Harb Protoc 2018:462–464. doi:10.1101/pdb.prot095489 29858336

[43] Altschul SF, Gish W, Miller W, Myers EW, Lipman DJ. 1990. Basic local alignment search tool. J Mol Biol 215:403–410. doi:10.1016/S0022-2836(05)80360-2 2231712

[44] Kumar S, Stecher G, Li M, Knyaz C, Tamura K. 2018. MEGA X: molecular evolutionary genetics analysis across computing platforms. Mol Biol Evol 35:1547–1549. doi:10.1093/molbev/msy096 29722887 PMC5967553

[45] Di Tommaso P, Moretti S, Xenarios I, Orobitg M, Montanyola A, Chang J-M, Taly J-F, Notredame C. 2011. T-Coffee: a web server for the multiple sequence alignment of protein and RNA sequences using structural information and homology extension. Nucleic Acids Res 39:W13–W17. doi:10.1093/nar/gkr245 21558174 PMC3125728

[46] Wilkins MR, Gasteiger E, Bairoch A, Sanchez JC, Williams KL, Appel RD, Hochstrasser DF. 1999. Protein identification and analysis tools in the ExPASy server. Methods Mol Biol 112:531–552. doi:10.1385/1-59259-584-7:531 10027275

[47] Gautier R, Douguet D, Antonny B, Drin G. 2008. HELIQUEST: a web server to screen sequences with specific α-helical properties. Bioinformatics 24:2101–2102. doi:10.1093/bioinformatics/btn392 18662927

[48] Drozdetskiy A, Cole C, Procter J, Barton GJ. 2015. JPred4: a protein secondary structure prediction server. Nucleic Acids Res 43:W389–W394. doi:10.1093/nar/gkv332 25883141 PMC4489285

[49] Jones DT. 1999. Protein secondary structure prediction based on position-specific scoring matrices. J Mol Biol 292:195–202. doi:10.1006/jmbi.1999.3091 10493868

[50] Geourjon C, Deléage G. 1995. SOPMA: significant improvements in protein secondary structure prediction by consensus prediction from multiple alignments. Comput Appl Biosci 11:681–684. doi:10.1093/bioinformatics/11.6.681 8808585

[51] Tieleman DP, Sansom MSP. 2001. Molecular dynamics simulations of antimicrobial peptides: from membrane binding to trans-membrane channels. Int J Quantum Chem 83:166–179. doi:10.1002/qua.1208

[52] Trott O, Olson AJ. 2010. AutoDock Vina: improving the speed and accuracy of docking with a new scoring function, efficient optimization, and multithreading. J Comput Chem 31:455–461. doi:10.1002/jcc.21334 19499576 PMC3041641

[53] Wang G. 2020. Bioinformatic analysis of 1000 amphibian antimicrobial peptides uncovers multiple length-dependent correlations for peptide design and prediction. Antibiotics 9:491. doi:10.3390/antibiotics9080491 32784626 PMC7459754

[54] Adade CM, Oliveira IRS, Pais JAR, Souto-Padrón T. 2013. Melittin peptide kills Trypanosoma cruzi parasites by inducing different cell death pathways. Toxicon 69:227–239. doi:10.1016/j.toxicon.2013.03.011 23562368

[55] Jacobs T, Bruhn H, Gaworski I, Fleischer B, Leippe M. 2003. NK-lysin and its shortened analog NK-2 exhibit potent activities against Trypanosoma cruzi. Antimicrob Agents Chemother 47:607–613. doi:10.1128/AAC.47.2.607-613.2003 12543667 PMC151766

[56] Pinto EG, Pimenta DC, Antoniazzi MM, Jared C, Tempone AG. 2013. Antimicrobial peptides isolated from Phyllomedusa nordestina (Amphibia) alter the permeability of plasma membrane of Leishmania and Trypanosoma cruzi. Exp Parasitol 135:655–660. doi:10.1016/j.exppara.2013.09.016 24113627

[57] Nagajyothi F, Machado FS, Burleigh BA, Jelicks LA, Scherer PE, Mukherjee S, Lisanti MP, Weiss LM, Garg NJ, Tanowitz HB. 2012. Mechanisms of Trypanosoma cruzi persistence in Chagas disease. Cell Microbiol 14:634–643. doi:10.1111/j.1462-5822.2012.01764.x 22309180 PMC3556388

[58] Eggimann GA, Sweeney K, Bolt HL, Rozatian N, Cobb SL, Denny PW. 2015. The role of phosphoglycans in the susceptibility of Leishmania mexicana to the temporin family of anti-microbial peptides. Molecules 20:2775–2785. doi:10.3390/molecules20022775 25668079 PMC6272152

[59] Kang HK, Seo CH, Park Y. 2015. Marine peptides and their anti-infective activities. Mar Drugs 13:618–654. doi:10.3390/md13010618 25603351 PMC4306955

[60] Valdivieso-Rivera F, Bermúdez-Puga S, Proaño-Bolaños C, Almeida JR. 2022. Deciphering the limitations and antibacterial mechanism of cruzioseptins. Int J Pept Res Ther 28:282. doi:10.1007/s10989-022-10383-4

[61] Cardoso MH, Ribeiro SM, Nolasco DO, de la Fuente-Núñez C, Felício MR, Gonçalves S, Matos CO, Liao LM, Santos NC, Hancock REW, Franco OL, Migliolo L. 2016. A polyalanine peptide derived from polar fish with anti-infectious activities. Sci Rep 6:21385. doi:10.1038/srep21385 26916401 PMC4768251

[62] Migliolo L, Felício MR, Cardoso MH, Silva ON, Xavier M-A, Nolasco DO, de Oliveira AS, Roca-Subira I, Vila Estape J, Teixeira LD, Freitas SM, Otero-Gonzalez AJ, Gonçalves S, Santos NC, Franco OL. 2016. Structural and functional evaluation of the palindromic alanine-rich antimicrobial peptide Pa-MAP2. Biochim Biophys Acta 1858:1488–1498. doi:10.1016/j.bbamem.2016.04.003 27063608

[63] Robles-Loaiza AA, Pinos-Tamayo EA, Mendes B, Ortega-Pila JA, Proaño-Bolaños C, Plisson F, Teixeira C, Gomes P, Almeida JR. 2022. Traditional and computational screening of non-toxic peptides and approaches to improving selectivity. Pharmaceuticals (Basel) 15:323. doi:10.3390/ph15030323 35337121 PMC8953747

